# Comment on “Back-to-base Versus In-transit Machine Perfusion in Donation After Circulatory Death Liver Transplantation: Insights From a National Registry”

**DOI:** 10.1097/TXD.0000000000001999

**Published:** 2026-09-09

**Authors:** David Walls, Shekhar Gogna, Piyush Gupta, Talal Al-Qaoud

**Affiliations:** 1 MedStar Georgetown Transplant Institute, MedStar Georgetown University Hospital, Washington, DC.

To the Editor:

We read with interest the recent article by Alderete et al^1^ comparing back-to-base and in-transit normothermic machine perfusion (NMP) for donation after circulatory death (DCD) livers. The authors are to be commended for a rigorous national analysis, which found comparable posttransplant outcomes between the 2 strategies. We raise several points, however, that we believe would strengthen the interpretation of these findings.

First, we would be interested to know whether the authors accounted for the use of normothermic regional perfusion (NRP) in the donor. A growing body of evidence indicates that NRP reduces ischemic cholangiopathy and improves graft survival in DCD liver transplantation, such that NRP-procured grafts can be transplanted with excellent outcomes without subsequent NMP.^2^ Beyond its direct effect on graft quality, prior NRP may also shape the choice of preservation strategy itself. A center may be more willing to accept a longer cold-ischemia time, and therefore a back-to-base model, for a graft that will undergo in situ reconditioning. Additionally, donor warm ischemia time is known to have a significant impact on graft outcomes^3^ and we wonder if this was examined between the 2 groups as an additional confounder.

Second, we would welcome a subgroup analysis in the grafts most vulnerable to cold-ischemic injury, particularly those from older donors, donors with a longer warm ischemia time, and those with a greater degree of steatosis.^4^ Any disadvantage of a back-to-base approach may be concentrated within these higher-risk grafts rather than evident across the cohort as a whole. We would also caution on the registry itself. As the authors acknowledge, the reliability of NMP reporting is limited: the cold-ischemia interval from cross-clamp to anastomosis encompasses both static cold storage and device time, leaving the true NMP duration unknown. Classifying every machine-perfusion-flagged graft as “in-transit” also risks misclassification, since back-to-base programs may report perfusion to UNOS. These gaps underscore the need for the OPTN to improve the granularity of machine-perfusion reporting.

Third, it is also worth acknowledging the inherent selection bias. Every included liver was deemed transplantable, having presumably satisfied viability criteria during NMP. As a result, comparable outcomes between the 2 models are not necessarily surprising especially without an analysis of ischemic cholangiopathy. The pertinent question that remains is whether the longer cold-ischemia time of a back-to-base approach leads to a greater number of discarded grafts that might have been successfully transplanted had in-transit perfusion been utilized.

Finally, these considerations converge on a central unresolved question: whether NRP can buffer the adverse effects of prolonged cold ischemia in DCD liver transplantation, and whether subsequent normothermic or hypothermic machine perfusion can further improve outcomes or, at minimum, provide additional time to identify a more suitable recipient. The eventual availability of portable hypothermic oxygenated perfusion^5^ makes its sequential use with a back-to-base workflow a compelling avenue, but these interactions should be addressed prospectively, ideally through randomized controlled trials.

We again thank the authors for this valuable contribution and look forward to further work in this rapidly evolving field.

D.W., S.G., P.G., and T.A. participated in the writing of the article.

The authors declare no funding or conflicts of interest.
